# The microRNA -23b/**-**27b Cluster Suppresses the Metastatic Phenotype of Castration-Resistant Prostate Cancer Cells

**DOI:** 10.1371/journal.pone.0052106

**Published:** 2012-12-26

**Authors:** Reema A. Ishteiwy, Toby M. Ward, Derek M. Dykxhoorn, Kerry L. Burnstein

**Affiliations:** 1 Department of Molecular and Cellular Pharmacology, Miller School of Medicine, University of Miami, Miami, Florida, United States of America; 2 John T. Macdonald Foundation Department of Human Genetics, Department of Microbiology and Immunology, John P. Hussman Institute for Human Genomics, Miller School of Medicine, University of Miami, Miami, Florida, United States of America; Roswell Park Cancer Institute, United States of America

## Abstract

MicroRNAs (miRs) are small, endogenous, non-coding RNAs that regulate the stability and/or translation of complementary mRNA targets. MiRs have emerged not only as critical modulators of normal physiologic processes, but their deregulation may significantly impact prostate and other cancers. The expression of miR-23b and miR-27b, which are encoded by the same miR cluster (miR-23b/-27b), are downregulated in metastatic, castration-resistant tumors compared to primary prostate cancer and benign tissue; however, their possible role in prostate cancer progression is unknown. We found that ectopic expression of miR-23b/-27b in two independent castration-resistant prostate cancer cell lines resulted in suppression of invasion and migration, as well as reduced survival in soft agar (a measure of anoikis). However, there was no effect of miR-23b/-27b on cell proliferation suggesting that these miRs function as metastasis (but not growth) suppressors in prostate cancer. Conversely, inhibition of miR-23b/-27b in the less aggressive androgen-dependent LNCaP prostate cancer cell line resulted in enhanced invasion and migration also without affecting proliferation. Mechanistically, we found that introduction of miR-23b/-27b in metastatic, castration-resistant prostate cancer cell lines resulted in a significant attenuation of Rac1 activity without affecting total Rac1 levels and caused increased levels of the tumor suppressor E-cadherin. Inhibition of these miRs had the opposite effect in androgen-dependent LNCaP cells. These results suggest that miR-23b/-27b are metastasis suppressors that might serve as novel biomarkers and therapeutic agents for castration-resistant disease.

## Introduction

For over half a century, androgen deprivation has been the standard therapy for advanced and metastatic prostate cancer, as tumors are initially dependent on androgens for survival and growth. Unfortunately, in most patients, tumors eventually progress to an incurable, castration-resistant and metastatic form. Hence, effective new therapies and accurate prognostic indicators are needed to improve clinical care of men with prostate cancer.

Metastasis, a hallmark of malignancy, is the migration of tumor cells via the bloodstream or lymph system from the original tumor site to distant organs. Metastatic development proceeds through a multistep process that includes local invasion, movement into the bloodstream (intravasation), survival in the circulation, exit from blood vessels (extravasation), initiation and maintenance of micrometastases at distant sites and finally, vascularization of the new tumors [1]. In order to proceed down this metastatic cascade, primary tumor cells accumulate genetic and epigenetic changes, including the deregulation of miRNA expression patterns. Elucidating the mechanisms that facilitate cancer cell migration and invasion is a major goal of cancer research, as metastasis remains the cause of 90% of deaths from solid tumors [1]. The median survival for patients with localized prostate cancer is greater than 5 years, whereas men with metastatic disease have substantially diminished survival rates [1].

MicroRNAs (miRs), small noncoding 18- to 24-nucleotide RNAs, are predicted to regulate expression of greater than 90% of protein encoding genes, thereby affecting diverse cellular and molecular processes [2]. MiRs modulate mRNA levels and translation through canonical base pairing between the seed sequence of the miRNA (nucleotides 2–8 at the 5′end) and the complementary seed match sequences of target mRNAs, which are typically located in the 3′ untranslated region (UTR) [3]. MicroRNAs silence their cognate targets by mRNA cleavage, translational repression, mRNA destabilization or a combination of these mechanisms [4]. More than 50% of annotated human miR genes are located in chromosomal regions that are susceptible to amplification, deletion or translocation during the course of tumor development [5]. MiRs play a critical role in metastasis, likely due to their ability to post-transcriptionally regulate gene networks important for cell invasion, motility and migration [6].

The biogenesis of miRs is a highly regulated, multistep process [7]. Over 40% of human miRs are organized in evolutionarily conserved clusters, which are cotranscribed as discrete polycistronic pri-miRNAs. A large number of miRs and miR clusters are deregulated in oncogenesis and metastatic development [8].

MiR-23b and miR-27b, which comprise a cluster on human chromosome 9, are specifically down-regulated in human castration-resistant prostate cancer (CRPC) clinical samples [9]–[12], as well as in cell models of CRPC [10], [13]. Interestingly, Sun et al. [12] found that miR-23b/-27b expression is significantly decreased (2.8-fold) in primary tumors (compared to adjacent normal tissue) and is further decreased by 3.2-fold in metastatic CRPC samples. In this study, 15 out of 17 CRPC tumor samples exhibited downregulation of miR-23b/-27b as compared to primary tumor samples [12]. Despite the correlation of decreased miR-23b/-27b with increased disease pathogenesis, the role of miR-23b/-27b in CRPC metastatic disease has not been well characterized. Here we provide evidence that miR-23b/-27b suppresses key metastatic processes including cell invasion, migration and anchorage-independent survival without affecting cell proliferation. These effects are accompanied by increased E-cadherin levels and attenuation of Rac1 activity.

E-cadherin, a cell adhesion molecule, suppresses the invasive and migratory phenotype of cancer cells [14]–[17]. Loss of E-cadherin is typically associated with tumor invasiveness, metastatic dissemination and poor patient prognosis in a variety of cancers, including prostate. For example, loss of E-cadherin confers metastatic ability to relatively non aggressive, transformed breast epithelial cells [16]. Furthermore, E-cadherin negative cells exhibit enhanced invasion and metastatic potential as compared to E-cadherin positive cells in the Dunning rat prostate tumor model [14], [15], [18]. The Rho GTPase Rac1, which regulates cytoskeleton rearrangements necessary for cell migration, is strongly associated with aggressive prostate cancer [19]–[22]. Elevated Rac1 is required for invasive behavior of the human prostate cancer cell line PC3 [23]. Thus, data presented here are consistent with a role for the miR-23b/-27b cluster in metastasis suppression via effects on E-cadherin and Rac1.

## Materials and Methods

### Cell Culture

The human prostate cancer cell lines ALVA31 (obtained from Drs. Stephen Loop and Richard Ostensen, Department of Veteran Affairs Medical Center, Tacoma, WA) [24], LNCaP (American Type Culture Collection, Manassas, VA), PC3-ML (obtained from Dr. Alessandro Fatatis, Drexel University College of Medicine) [25] were passaged and maintained in RPMI 1640 medium supplemented with 10% fetal bovine serum, 100 IU/ml penicillin, 100 g/ml streptomycin, and 100 g/ml L-glutamine. All of the cultures were maintained at 37°C in a humidified atmosphere of 5% CO_2._

### RNA Isolation and Quantitative RT-PCR

Total RNA was isolated using miRVana miRNA isolation kit (Applied Biosystems, Foster City, California). The TaqMan stem-loop RT-PCR method using the Taqman® miRNA reverse transcription kit and the Taqman® miRNA assays (Applied Biosystems) was used to assess the expression of miR-27b.U6 small nuclear RNA served as an internal control for all experiments.

### Immunoblotting

Cellular proteins were extracted and separated on SDS-PAGE gels, and western blot analyses were performed according to standard procedures. Western blotting of β-actin on the same membrane was used as a loading control. The antibodies used were anti-E-cadherin (BD 610181), anti-Rac1 (Milipore 23A8), and anti-actin (Santa Cruz 1616).

### Plasmids and Lentiviral Production

The human miR-23b/-27b precursor (pMIRNA-23b/-27b-GFP) and scrambled control (pMIRNA-Scrambled-GFP) cloned into lentiviral vectors (Systems Biosciences (SBI) Mountain View CA, USA) were transfected into LentiX HEK cells (Clontech, Mountain View CA, USA) according to the manufacturer’s protocol. GFP expression in transduced ALVA31 and PC3-ML cells was determined by flow cytometery.

### Flow Cytometry

Trypsinized cells were permeabilized using 0.05% Trypsin with 0.53 mM EDTA (Cellgro). Flow cytometry was performed on an Accuri C6 cytometry using CFlow software according to the manufacturer’s instructions.

### Cell Transfections

Chemically modified antisense nucleotides (antagomiRs) against miR-23b and miR-27b or control antagomiRs were purchased from Applied Biosystems and transfected at 50 nM using Lipofectamine 2000 (Invitrogen) according to the manufacturer’s protocol. All experimental assays were performed 72 hours post transfection.

### Migration Assays

Scratch assays were performed on ALVA31 cells 72 hours after transduction with miR-23b/-27b or scrambled control or on LNCaP cells 72 hours after transfection with antagomiR-23b/-27b or control antagomiR. A sterile pipette tip was dragged across confluent cell monolayers to create scratch. Monolayers were then washed twice to remove cell debris and incubated at 37°C 5% CO_2_ for 4 hours. Photographs were taken immediately after scratching and again after 4 hours. Image J was used to measure scratch width as a function of time. The percent of gap (scratch) closure was calculated as the percentage of the remaining cell free area as compared to the area of the initial wound. Two independent experiments were performed with a total of at least 6 scratches for each cell line.

### Cell Proliferation Assays

ALVA31, PC3-ML (expressing miR-23b/-27b or scrambled control) and LNCaP cells (transfected with antagomiR-23b/-27b or control antagomiR) were plated at an initial density of 10,000 cells/well in a six-well plate. At the indicated time points, the cells were trypsinized and viable cells (those that exclude trypan blue) were counted using a hemocytometer.

### Matrigel Invasion Assays

Matrigel Invasion Assay (BD Biosciences) were performed using ALVA31 and PC3-ML cells 72 hours after transduction with miR-23b/-27b or scrambled control and LNCaP cells 72 hours after transfection with antagomiR-23b/-27b or control antagomiR. 50,000 cells were serum starved for 16 hours then seeded into the top chamber of the transwell apparatus with the Matrigel coated membrane (24 well insert, 8mm pore size). Medium supplemented with 10% FBS was used as chemoattractant. After incubation at 37°C for 48 hours, the top chambers were wiped with cotton wool to remove non-migratory or non-invasive cells. Cells on the lower surface of the membrane were then fixed with cold methanol, stained with 0.01% crystal violet and counted under a microscope.

### Soft Agar Assays

Soft agar assays were performed on ALVA31 or PC3-ML cells 72 hours after transduction with miR-23b/-27b or scrambled control. Anchorage-independent growth was evaluated as previously described [26].

### Rac1 Activity Assays

Rac1 activity assays were performed on ALVA31 or PC3-ML cells 72 hours after transduction with miR-23b/-27b or scrambled control. GTP-bound Rac1 was separated from GDP-Rac1 using a pull-down approach as previously described [19]. Briefly, cell lysates were incubated with 200 mg/ml PBD-GST [p21-binding domain, PBD, of the Rac1/Cdc42 effector PAK (p21-activated kinase)]. GTP-Rac1 was recovered following incubation with glutathione-agarose beads. Complexes were collected, denatured and resolved by SDS-PAGE. Active Rac1 was detected by Western blotting with anti-Rac1 antibodies (Millipore). A total of 5% of the original lysate was also analyzed by immunoblot to determine total levels of Rac1 (GDP-and GTP-bound).

## Results

### MiR-23b/-27b Suppresses Motility, Invasion and Anchorage-independent Growth of Castration-resistant Prostate Cancer Cells

Given the inverse correlation between miR-23b/-27b expression and malignant phenotype of human prostate cancer, we sought to assess the potential anti-neoplastic role(s) for this miRNA cluster. To that end, the miR-23b/-27b cluster was ectopically expressed using a lentiviral vector (also encoding GFP) in two independent highly aggressive castration-resistant prostate cancer cell lines, ALVA31 and PC3-ML. 72 hours following transduction, cells were analyzed for GFP expression using flow cytometry in order to determine transduction efficiency for each experiment (S1). Experiments were performed only when greater than 95% of cells expressed GFP. Since miR-27b is located 3′ to miR-23b, we monitored miR-27b levels by reverse transcriptase qPCR following transduction with lentiviruses encoding this miRNA cluster or a control non-specific miRNA (data not shown). Transduction with the miR-23b/-27b lentiviral vector resulted in mir-27b levels comparable to those naturally found in MCF7 cells, which served as a positive control for miR-27b [27]. Alternatively, miR-23b/-27b was inhibited in the androgen-dependent LNCaP line using antagomiRs, which are synthetic oligonucleotides complementary to the target miRNA that lead to cleavage of the corresponding miRNA (S2).

**Figure 1:**
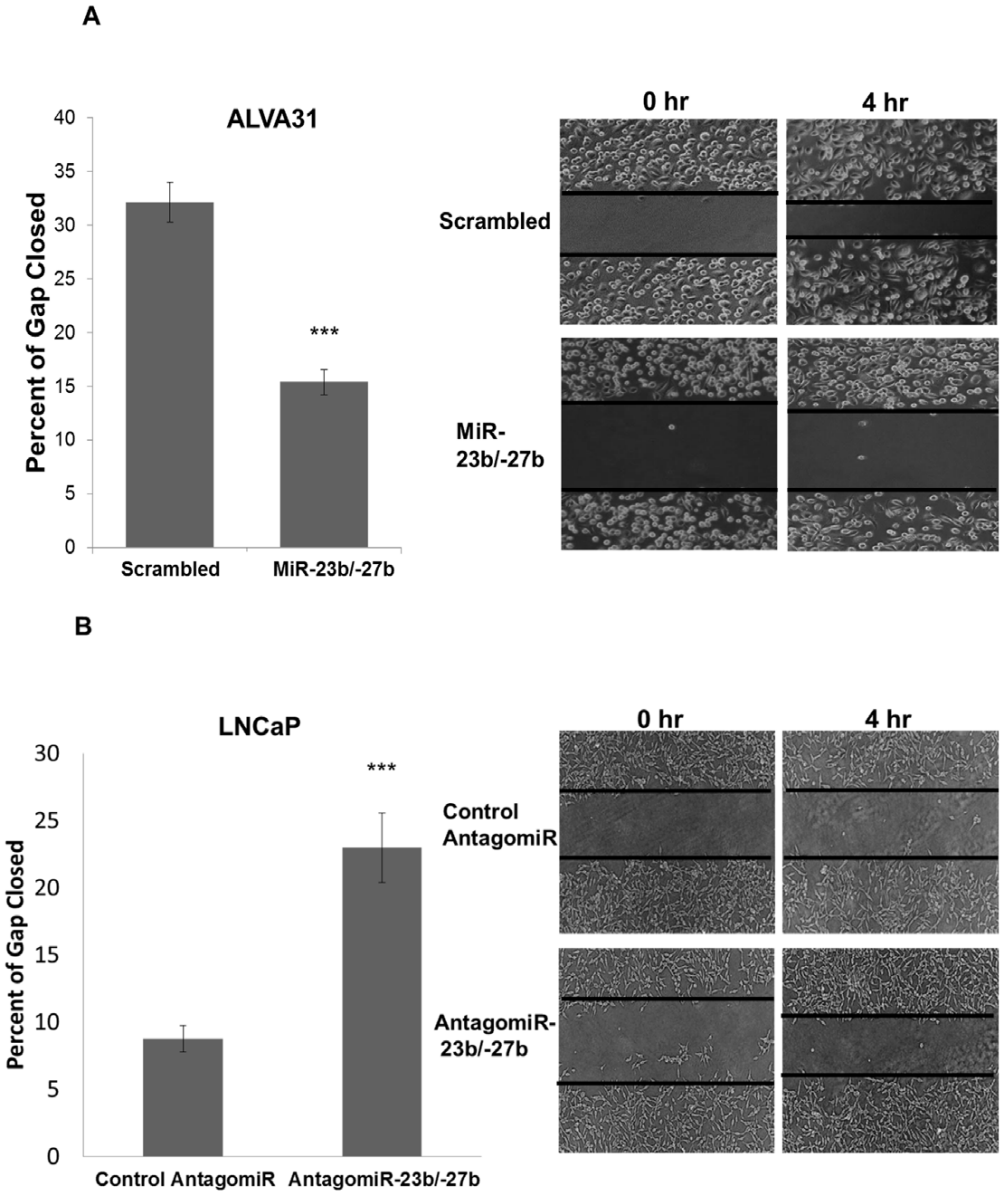
MiR-23b/ -**27b expression decreases castration-resistant prostate cancer cell migration while inhibition of miR-23b/**-**27b increases migration of androgen-dependent prostate cancer cells.** Scratch assays were performed on ALVA31 cells expressing miR-23b/-27b or scrambled control (A) and LNCaP cells transfected with 50 nM antagomiR-23b-27b or control antagomiR (B). Images of the cleared zones (representative images shown on the right) were taken before and after a 4 hr incubation and the cleared areas measured using Image J software. The mean percentage of gap closure (±SD) (due to cell migration) is shown for two independent experiments from 7 scratches (A) and 6 scratches (B) (***P<0.001).

To determine whether miR-23b/-27b regulates cellular metastatic processes, we analyzed the effects of altering miR-23b/-27b expression on castration-resistant cell motility and migration. Using scratch assays, we found that ectopic expression of miR-23b/-27b in the CRPC line, ALVA31 decreased cell motility approximately 2-fold after only 4 hours (Figure 1A). Conversely, inhibition of endogenous miR-23b/-27b expression in LNCaP cells, which have relatively high endogenous levels of these miRNAs and exhibit little motility, resulted in greater than 2-fold increased motility as compared to control antagomiR-transfected cells (Figure 1B).

**Figure 2:**
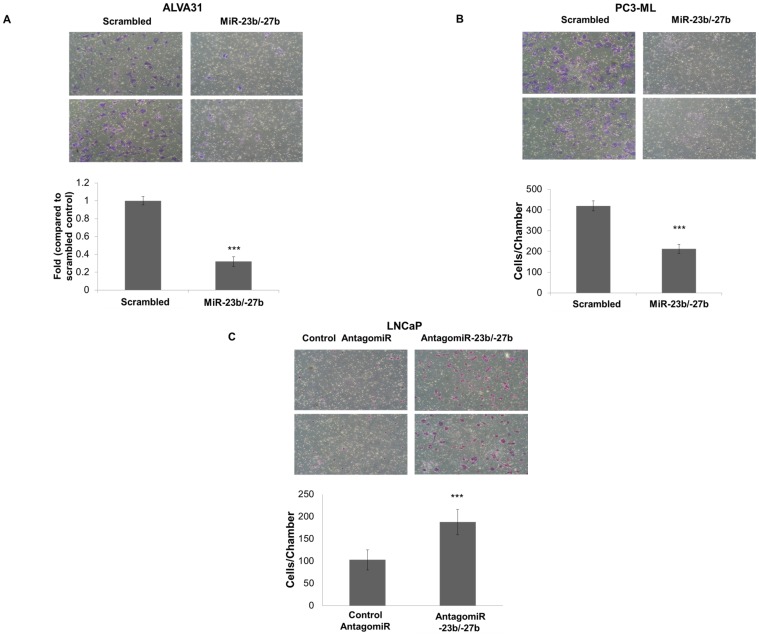
MiR-23b/ -**27b expression decreases the invasiveness of castration-resistant prostate cancer cells while inhibition of miR-23b/**-**27b in androgen-dependent prostate cancer cells increases invasiveness.** A, Matrigel invasion assays were performed on ALVA31 cells (A) and PC3-ML cells (B) expressing miR-23b/-27b or scrambled control-transduced cells. Upper panels show representative regions of the chamber filters with crystal violet-stained cells. The fold change (±SEM) represents the number of invaded cells per chamber divided by controls from three independent experiments performed in triplicate for A and the means (±SD) of one independent experiment done in triplicate for B (***P<0.001). C, LNCaP cells were transfected with 50 nM control antagomiR or antagomiR-23b/-27b. Matrigel invasion assays were performed 72 hours post transfection as explained above. The means (±SD) of two independent experiments performed in triplicate are shown (***P<0.001).

Next, we determined whether miR-23b/-27b expression affected invasiveness of castration-resistant cells using Matrigel invasion chambers. Serum starved cells were added to upper chambers of the transwell and the numbers of cells invading through the Matrigel barrier in response to chemoattractant (serum) during a 48 h time period were assessed. Introduction of miR-23b/-27b into ALVA31 and PC3-ML cells significantly decreased the number of invading cells by more than 2-fold (Figure 2A and B). In contrast, depletion of miR-23b/-27b activity in the less aggressive androgen-dependent LNCaP cell line significantly enhanced cell invasion (Figure 2C).

**Figure 3:**
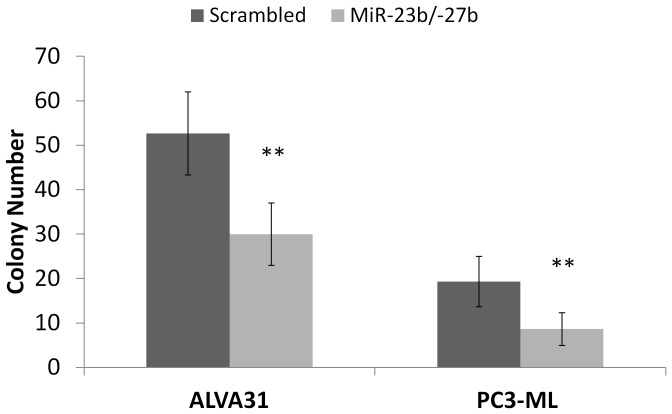
MiR-23b/ -**27b decreases anchorage independent growth of castration-resistant prostate cancer cell lines.** Soft agar assays were performed on ALVA31 and PC3-ML cells expressing miR-23b/-27b or transduced with scrambled control (**P<0.01). Mean colony number (±SEM) per plate from two independent experiments performed in triplicate for each cell line is shown (**P<0.01).

In addition to migration and invasion, metastatic carcinoma cells often acquire the capacity to grow in an anchorage-independent manner, resisting anoikis. Hence, we assayed the effects of miR-23b/-27b expression on prostate cancer cell survival in soft agar. ALVA31 and PC3-ML cells (miR-23b/-27b expressing and scrambled control transduced) were plated on low-attachment media (soft agar) and colony survival was evaluated 2–4 weeks after plating. Colony formation was significantly impaired in ALVA31 and P3-ML cell lines expressing miR-23b/-27b compared to the scrambled controls (Figure 3). These data strongly suggest that miR-23b/-27b expression confers anoikis sensitivity to aggressive castration-resistant prostate cancer cells.

**Figure 4:**
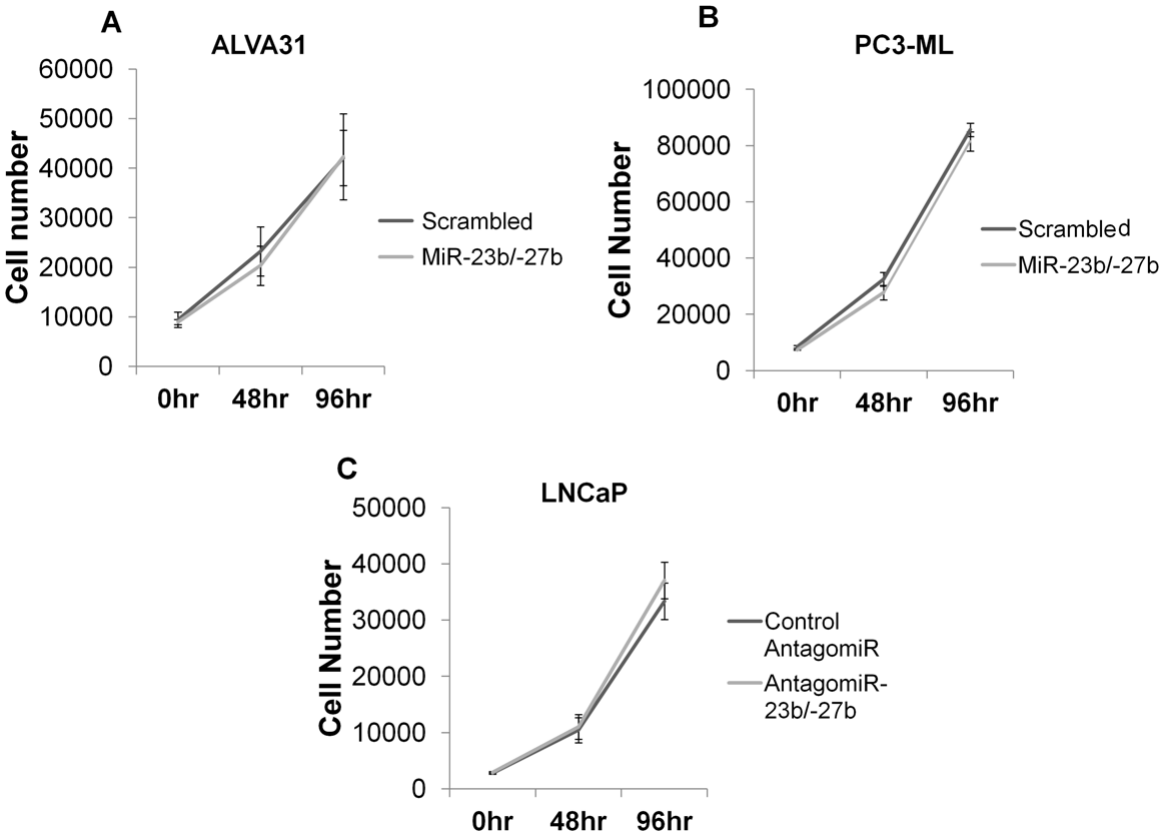
MiR-23b/ -**27b does not regulate prostate cancer cell proliferation.** A and B, Proliferation of cells expressing miR-23b/-27b was compared to that of scrambled control-transduced cells. The mean cell number (±SEM) of a total of three independent experiments performed in triplicate is shown for ALVA31 cells and the mean cell number (±SD) of a representative experiment performed in triplicate is shown for PC3-ML cells. C, Proliferation of LNCaP cells transfected with antagomiR-23b/-27b or control antagomiR was assessed. The mean cell number (±SD) from two independent experiments performed in triplicate is shown.

Interestingly, over-expression of miR-23b/-27b in the castration-resistant cell lines, ALVA31 and P3-ML, did not significantly affect cell proliferation (Figure 4A and B). Furthermore, inhibition of these miRs in androgen-dependent LNCaP prostate cancer cells also did not affect proliferation rates (Figure 4C). These results suggest that the miR-23b/-27b cluster may play a key role in the metastatic process, as indicated by the observed phenotypes in invasion, migration and anoikis assays. However, alterations in the level of the miR-23b/-27b cluster had no effect on proliferation of prostate cancer cell lines.

### Ectopic Expression of miR-23b/-27b Markedly Increases E-cadherin Protein Levels and Reduces Rac1 Activity

**Figure 5:**
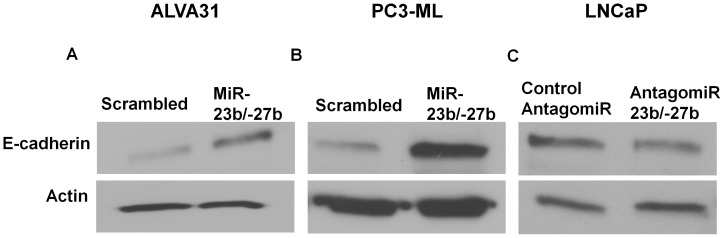
MiR-23b/ -**27b significantly increases E-cadherin expression in castration –resistant cell lines.** ALVA31 or PC3-ML cells expressing miR-23b/-27b or transduced with scrambled control and LNCaP cells transfected with antagomiR-23b/-27b or control antagomiR were subjected to western blotting for E-cadherin and actin. Representative blots are shown of a total of 2–6 experiments.

To understand the molecular mechanisms of miR-23b/-27b mediated inhibition of metastatic phenotypes, we examined the expression of key factors in cancer cell migration and invasion. Typically, migration and invasion of tumor cells is promoted by the loss of interaction of adherens junctions with the cytoskeleton and subsequent changes in the activities of Rho family small GTPases such as Rac1 and Cdc42 [28]. We found that ectopic expression of miR-23b/-27b resulted in a marked increase of E-cadherin protein levels in both ALVA31 and PC3-ML cells. Conversely, inhibition of miR-23b/-27b in LNCaP cells resulted in decreased E-cadherin levels (Figure 5).

**Figure 6:**
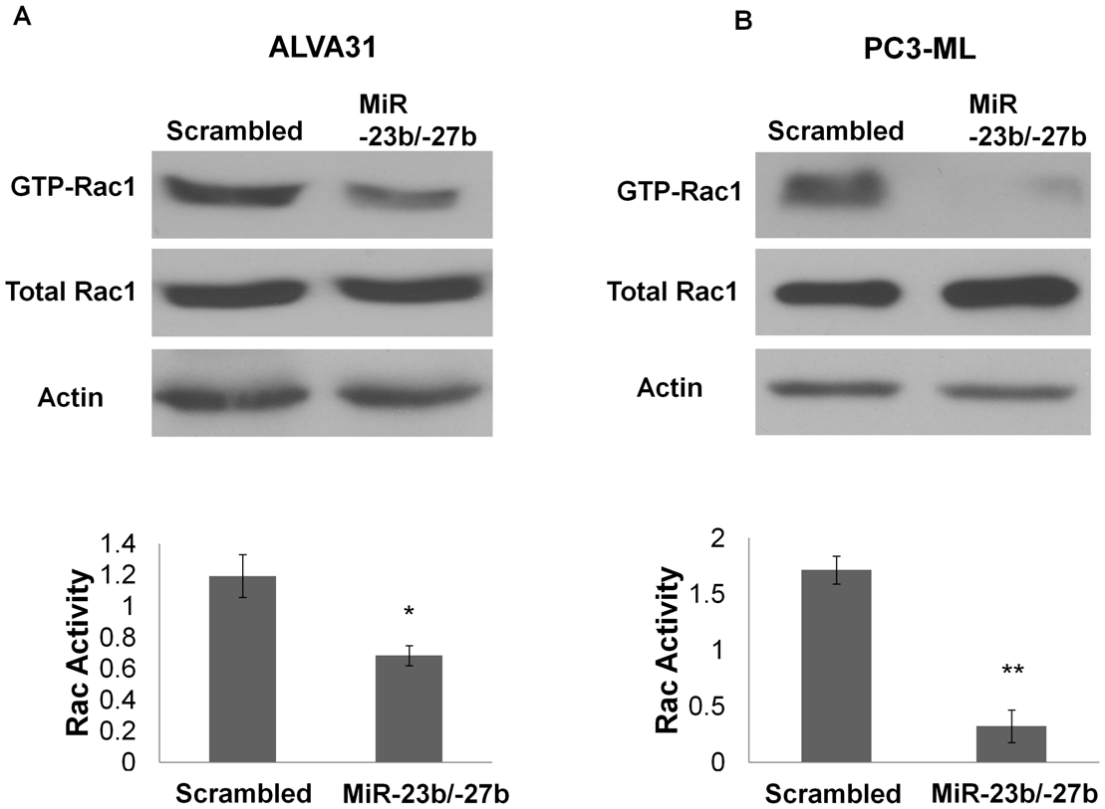
MiR-23b/ -**27b significantly decreases Rac1 activity but not total Rac1 levels in castration-resistant prostate cancer cell lines.** Rac1 activity assays were performed on ALVA31 (A) and PC3-ML (B) cells expressing miR-23b/-27b or transduced with scrambled control. GTP-bound Rac1 was separated from GDP-Rac1 using a pull-down assay as described in Materials and Methods. Complexes containing GTP-Rac1 (active Rac1) were collected, denatured and resolved by SDS-PAGE followed by Western blotting with anti-Rac1 antibodies. Total Rac1 (GDP-and GTP-bound) represents 5% of the original cell lysate. Actin was used as a loading control. Quantification of three independent experiments is shown with error bars representing SEM (*P<0.05), (**P<0.01).

Rac1 activity is associated with and appears to be essential for the metastatic phenotype of prostate cancer, as well as, other epithelial cancers [19], [29]. Endogenous Rac1 activity promotes anchorage independent growth and is required for invasive behavior of the CRPC cell line PC3 [29]. For these reasons, we determined whether ectopic expression of miR-23b/-27b in aggressive prostate cancer cells affected Rac1 activity. Introduction of miR-23b/-27b in ALVA31 and PC3-ML, both of which have high endogenous Rac1 activity [19], significantly attenuated Rac1 activity without affecting total Rac1 levels (Figure 6). Taken together, these data provide mechanistic insight into the role of miR-23b/-27b in suppression of metastatic phenotypes.

## Discussion

MiRs modulate a wide variety of biological and pathologic processes including the metastatic cascade. Over a thousand miRs are encoded in the human genome and the elucidation of their functions is an important research area [6]. Since many miRs have roles in apoptosis and mitogenesis [30] their potential contribution to metastasis can be difficult to discern. We show here that miR-23b/-27b suppressed migration and invasion of aggressive prostate cancer cells without exerting confounding influences on cellular proliferation. MiRs with these types of characteristics are described as candidate “metastasis suppressors” [6], [31].

The capacity of tumor cells to counteract anoikis, migrate and invade requires the acquisition of multiple molecular traits [32]. Our data show that ectopic expression of miR-23b/-27b in metastatic CRPC cell lines, ALVA31 and PC3-ML, resulted in decreased growth in soft agar as well as decreased cell migration and invasion. Conversely, inhibition of miR-23b/-27b in the androgen-dependent prostate cancer cell line, LNCaP, resulted in enhanced cell migration and invasion. In order for tumor epithelial cells to invade and migrate, the integrity of the epithelium and basement membrane must be disrupted allowing the cells to gain access into the underlying stroma. This process requires disruption of epithelial cell-cell contacts, which may occur through decreased levels of cell adhesion molecules such as E-cadherin. Expression of miR-23b/-27b increased E-cadherin protein levels in two independent CRPC cell lines; conversely inhibition of miR-23b/-27b in a less aggressive androgen-dependent prostate cancer cell line, LNCaP, resulted in a decrease of E-cadherin levels.

Loss of the cell adhesion molecule E-cadherin in prostate cancer patient specimens is strongly associated with metastatic behavior and poor clinical outcome [14], [15], [18]. These studies support E-cadherin deregulation as a critical event in prostate cancer progression and are consistent with our findings that miR-23b/-27b-expression resulted in increased E-cadherin levels and a decrease in metastatic traits. The molecular basis for up-regulation of E-cadherin in miR-23b/27b expressing prostate cancer cells is unknown. While we speculated that this effect is due to miR-23b/-27b-mediated inhibition (either direct or indirect) of one or several of the known E-cadherin transcriptional repressors [33]–[35], we did not observe consistent evidence of repression of the E-cadherin repressors Twist, Slug, Snail, Zeb1 or Zeb2 in CRPC cell lines overexpressing miR-23b/-27b (data not shown). These findings suggest the possible involvement of other repressors and/or that miR-23b/-27b-mediated up regulation of E-cadherin occurs through a more complex mechanism(s).

The metastatic process not only involves loss of cell-cell adhesions but tumor cells must also acquire the capacity to migrate in order to colonize distant sites. The Rho GTPase Rac1 regulates rearrangement of the actin cytoskeleton required for cell migration. Overexpression of Rac1 is associated with enhanced metastatic potential of prostate and other epithelial cancer types [36]–[38]. Elevated Rac1 activity occurs in CRPC, promotes castration resistant, as well as, anchorage independent growth and is required for invasive behavior of the CRPC cell line PC3 [19], [21]. A variety of activators of Rac1 have been shown to be important in prostate cancer progression or in the acquisition of an invasive phenotype. For example, we and others demonstrated the importance of Rho guanine nucleotide exchange factors (GEFs) in the progression of CRPC [21], [22], [26], [39]. Additionally, increased expression of certain Rac1 GEFs correlates with increased biochemical recurrence [22] and decreased disease free survival in prostate cancer patients [40]. Proteins that decrease Rac1 activity through hydrolysis of GTP, GTPase activating proteins (GAPs), act as tumor suppressors [41]. Although miR-23b/-27b did not affect levels of total Rac1, introduction of these miRs into ALVA31 and PC3-ML cells reduced levels of active (GTP-bound) Rac1. Therefore we presume that miR-23-b/27-b must inhibit one or more of the many upstream activators of Rac1 or indirectly increase Rac1 inhibitory proteins.

While expression of miR-23b/-27b mediated the reduction of Rac1 activity and resulted in increased E-cadherin protein levels, these effects are suspected to be indirect. Since individual miRs often regulate numerous target genes, miR-23b/-27b may repress multiple targets and networks that ultimately result in the major phenotypic changes that we observed. A number of miR-23b and miR-27b target genes have been identified in other cancer cell types in which these miRs are differentially expressed compared to normal tissue, including breast, liver and lung [42]–[50]. However, since many of the proteins encoded by these gene targets are involved in regulating cell proliferation, we anticipate that these genes do not represent the relevant targets in prostate cancer cells. In addition, we identified numerous computationally predicted candidate targets of miR-23b/-27b that are linked to Rac1 signaling and E-cadherin regulation including PI3-kinase, MAPK, TGF-Beta, Wnt, mTOR, Jak-STAT, toll-like receptor and Notch among others. Within these complex integrative networks, targets of miR-23b/-27b may converge to regulate Rac1 activity and E-cadherin. We are actively pursuing these possible targets. In addition, it will be important to delineate the individual roles of the two miRs in this cluster on invasion and migration of prostate cancer cells.

Since metastases are responsible for patient mortality in almost all human carcinomas, the ability of miR-23b/-27b to impede metastatic processes could be of clinical value. Loss of miR-23b/-27b may serve as a prognostic marker for aggressive prostate cancer. Biomarkers that distinguish indolent from aggressive prostate cancer are critically needed, and loss of miR-23b/-27b may predict metastatic disease. Since miRs also have potential use as therapeutic modalities [51], miR-23b/-27b “mimics” should be evaluated in preclinical models of prostate cancer metastasis.

## Supporting Information

Slide S1 **Assessment of transduction efficiency of ALVA31 and PC3-ML cells.** GFP expression of ALVA31 (A) or PC3-ML (B) cells 72 hours after two sequential transductions with the GFP-encoded lentiviral vectors pMIRNA-miR-23b/-27b or pMIRNA-scrambled lentiviral vectors (Systems Biosciences (SBI) Mountain View CA, USA). GFP expression in untransduced and transduced cells was assessed by FACS analysis.
(TIF)

Slide S2 **MiR-27b levels decrease in LNCaP cells transfected with antagomiR-23b/-27b.** LNCaP cells were transfected with 50 nM antagomiR-23b/-27b or control antagomiR. MiR-27b levels were determined using real time-PCR 72 hours following transfection. Values are the miR-27b level normalized to U6 snRNA in LNCaP cells transfected with control antagomiR.
(TIF)
