# Supplementary figures and images for: The microRNA -23b/-27b Cluster Suppresses the Metastatic Phenotype of Castration-Resistant Prostate Cancer Cells
Source: PLoS One. 2012 Dec 26;7(12):e52106. doi: 10.1371/journal.pone.0052106 (PMC3530545; doi:10.1371/journal.pone.0052106)

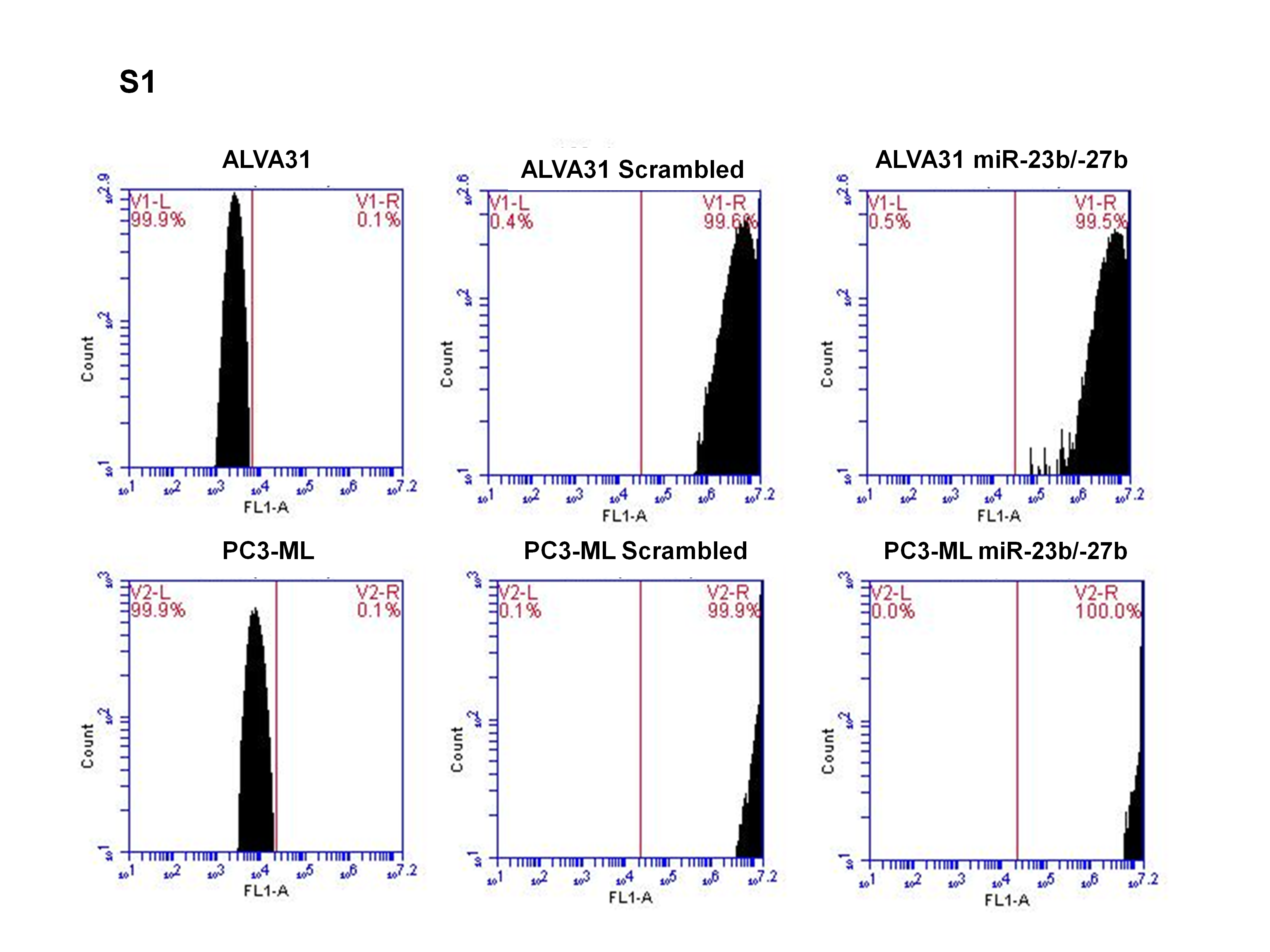

Supplement: Slide S1 — Assessment of transduction efficiency of ALVA31 and PC3-ML cells. GFP expression of ALVA31 (A) or PC3-ML (B) cells 72 hours after two sequential transductions with the GFP-encoded lentiviral vectors pMIRNA-miR-23b/-27b or pMIRNA-scrambled lentiviral vectors (Systems Biosciences (SBI) Mountain View CA, USA). GFP expression in untransduced and transduced cells was assessed by FACS analysis. (TIF) [file pone.0052106.s001.tif]
